# Trends of mastectomy and breast-conserving surgery and related factors in female breast cancer patients treated at King Abdulaziz University Hospital, Jeddah, Saudi Arabia, 2009–2017: A retrospective cohort study

**DOI:** 10.1016/j.amsu.2019.03.012

**Published:** 2019-04-02

**Authors:** Zuhoor K. Al –Gaithy, Bassam E. Yaghmoor, Mohammed I. Koumu, Khalid A. Alshehri, Abed A. Saqah, Hisham Z. Alshehri

**Affiliations:** a General Surgery and Oncoplastic Breast Surgeon, King Abdulaziz University Hospital, Jeddah, Saudi Arabia; b Faculty of Medicine, King Abdulaziz University, Jeddah, Saudi Arabia

**Keywords:** Breast surgery, Procedure choice, Mastectomy risk, Surgical outcome, Breast reconstruction

## Abstract

**Background:**

Breast cancer is the most common cancer in women and accounts for 14.7% of cancer-related deaths among females worldwide. Its core management includes surgical removal of the tumor either by breast-conserving surgery (BCS) or mastectomy. Choosing between these two procedures may be influenced by factors that are not studied in our region. We aimed to determine the prevalence of BCS and mastectomy and the factors that may influence the choice of procedure.

**Methods:**

This retrospective study was carried out by reviewing the records of female breast cancer patients who underwent BCS or mastectomy at between 2009 to June 2017, excluding those with metastasis or recurrence. Frequencies and multivariate tests were used for detecting correlations between procedures and demographic, clinicopathological, and radiological factors.

**Results:**

Of 335 patients (mean age 52.75 ± 12.2 years), 62.4% had mastectomy and 37.6% had BCS. Modified radical mastectomy accounted for 70.8% of mastectomies. Multivariate analysis showed non-Saudi nationality (P = 0.002), multifocal (P = 0.0001) and multicentric tumors (P = 0.0001), large tumor size (P = 0.0001), tumor stages IIIA (P = 0.005) and IIIB (P = 0.014), positive HER2 (0.009), and triple-negative receptor status (P = 0.010) significantly correlated with mastectomy.

**Conclusion:**

Mastectomy has a much higher prevalence than BCS in our study mainly due to advanced tumor stage at the time of diagnosis. This emphasizes the urgent need for early detection of breast cancer to move towards BCS, with education and increasing awareness of breast cancer and the surgical options, especially that it is more common in a significantly younger population in our area.

## Highlights

• Breast cancer's effect on the developing world is higher due to the late diagnosis.
• Predictors of mastectomy included increased size and stage and HER2 positivity.
• Mastectomy had a much higher prevalence than in the US and Europe.
• Awareness of breast cancer for early diagnosis and the surgical options is important.

## 1 Introduction

Breast cancer is the most common cancer in women across the world, accounting for 25.2% of cancer cases among females and 14.7% of cancer-related deaths among females worldwide [1]. Its effect on the developing world is more pronounced due to the late diagnosis and lack of resources and education. The survival rates are markedly lower in the developing countries compared to developed countries (80% vs. less than 40%), and consequent deaths in developing countries account for 58% of all breast cancer deaths worldwide [1,2]. In 2013, Saudi Arabia had an overall age-standardized breast cancer incidence rate of 25.5/100,000 women, according to the Saudi Cancer Registry [3]. Makkah region has a rate of 25.3/100,000 [4]. Many studies showed that the most commonly diagnosed group is females in their thirties and forties [3,5,6], which indicates an increasing pattern in Saudi Arabia. The pattern of early occurrence and late discovery is seen throughout the Arab World [7].

The core of breast cancer management includes removal of the tumor either by mastectomy or breast-conserving surgery (BCS; lumpectomy). Mastectomy has been the most common treatment in the field, but the emergence of BCS has triggered a shift. Radical mastectomy was first popularized by William Halsted in 1894 and resulted in a significant decline in the local recurrence rate, but the curative potential remained limited [8]. Subsequently, modified radical mastectomy [9] and skin-sparing mastectomy or subcutaneous mastectomy [10,11] were introduced. The attempt to maintain the breast without compromising survival led to the use of BCS [12]. Many prospective randomized trials proved that patient survival after undergoing BCS was similar to mastectomy in the treatment of invasive breast cancer. BCS has not only afforded an acceptable oncological outcome but also reduced the psychological burden, provided better cosmetic results, and decreased postoperative complications [12].

Regarding the prevalence of mastectomy and BCS, an institutional review board in the United States that included 5865 patients (5833 female, 32 male) from 1994 to 2007 reported that the mastectomy rate increased slowly during the study period (33%–44%) [13]. A cohort study in the USA consisted of 21,869 patients who underwent BCS or mastectomy as primary surgical therapy for stage 0, I or II breast cancer from 1998 to 2007 reported increases in the rate of using mastectomy for early-stage breast cancer treatment in all age groups [14]. Another large, long-term study from China on 4211 female patients between 1999 and 2008 found a more widespread use of mastectomy, as 94.3% underwent this procedure; however, there was an increase in BCS and decline in mastectomy use by 11% throughout the years [15].

Many factors influence the decision between these two procedures. Several studies suggested that increases in tumor size, metastasis to lymph nodes [16], pathologic stage and histologic grade all are linked to choosing mastectomy. An additional study in 2012 demonstrated the relation between mastectomy and age ≥70 years, living in a rural area, hormone receptor-negative tumor, axillary lymphadenectomy and lobular carcinoma, in addition to the factors in the previous studies [15]. Receiving chemotherapy preoperatively and the type of surgeon (either breast surgeon and neoplastic breast surgeons or general surgeon) were important factors that led to choosing BCS [17]. As a part of patients’ preference, having a discussion with the surgeon about breast reconstruction increases the likelihood of the patients choosing mastectomy although reconstruction is mostly not discussed [18]. Furthermore, using preoperative breast magnetic resonance imaging (MRI) has been found to have a high predictive value for the type of surgery. A retrospective cohort study conducted to assess this relationship found that preoperative breast MRI was correlated with higher mastectomy rates [19]. However, another study suggested that preoperative MRI is not always related to mastectomy, as some patients who were originally considering mastectomy chose BCS after MRI [20].

Regarding the outcomes of mastectomy and BCS, two large studies in the United States on female breast cancer patients with tumor size <5 cm found no significant difference in survival rate between the procedures [21,22]. In contrast, a study in 2013 showed better survival in the BCS patients [23]. Furthermore, performing mastectomy is correlated with low satisfaction, worse body image and negative impact on sexual life [24,25]. Such findings are in favor of BCS over mastectomy, especially since the peak incidence in Saudi Arabia is among younger women.

This retrospective study aimed to investigate the rate of mastectomy and breast-conserving surgery and related factors to determine whether they play a role in the choice of surgery in the female breast cancer patients at King Abdulaziz University Hospital (KAUH).

## 2 Materials and methods

### 2.1 Study design

A non-interventional, retrospective cohort study was conducted, in line with STROCSS [26] criteria.

### 2.2 Study setting

The study was conducted by reviewing the records of female breast cancer patients in the period between 2009 and 2017 who underwent BCS or mastectomy at one of the largest referral and academic medical centers in the western region of Saudi Arabia.

### 2.3 Participants

All female breast cancer patients in the period between 2009 and 2017 who underwent BCS or mastectomy in the hospital were included in our study (380 patients). All patients were under the general surgical unit, and all surgeries were carried out by general surgeons with no special training in breast cancer surgery. Breast cancer diagnosis was based on histopathology reports from biopsies. Exclusion criteria were as follows: patients who had the surgery outside our hospital, patients with metastatic or recurrent breast cancer, pregnant women, and patients who had prophylactic mastectomies. In bilateral breast cancer cases, each breast was recorded as a separate case.

### 2.4 Variables

The data collection was carried out by filling a data sheet that consisted of three parts. The first part was the demographic data such as the age at time of surgery, as well as nationality. The second part concerned the operation, including the type of surgery, year of operation, administration of neoadjuvant chemotherapy (NACT), immediate breast reconstruction after tumor removal, and reason for the choice of surgery if available. The third part was the histopathological and radiological nature of the tumors, largest diameter, multifocality, multicentricity, highest grade, lymph node involvement, receptor status, tumor lymphovascular invasion, and use of preoperative breast MRI. Size, multifocality, and multicentricity were recorded from imaging studies prior to surgery or, if not available, from histopathology reports. The use of preoperative MRI was done for patients who were candidates for NACT, had breast implants, or presented with axillary lymphadenopathy on clinical examination but with negative mammograms and ultrasound studies. It was also done when there was a suspicion of multifocality or multicentricity, especially in young patients or with positive family history of breast cancer.

The stages of the tumor were written later according to the American Joint Committee on Cancer Manual, 7th Edition [27].

### 2.5 Bias

The selection bias was been reduced by including all the female patients.

### 2.6 Confidentiality and ethical approval

All identifying variables of participants were removed so only anonymous data were used to ensure privacy and confidentiality. Only the investigators has access to the data. Approval of the Research Ethics Committee (REC) of the Faculty of Medicine was obtained.

### 2.7 Statistical analysis

Data were coded, checked, and entered into IBM SPSS Statistics for Windows, version 23 (IBM Corp., Armonk, N.Y., USA). Categorical variables, including primary variables, were described using frequencies. Continuous variables for normally distributed data were described using means and standard deviations. For categorical variables, odds ratios and their 95% confidence intervals (CIs) were estimated, and the Chi-square and Fisher-exact tests were performed. A P-value less than 0.05 was considered significant. To control for potential confounders, multivariate regression was used.

## 3 Results

**Fig. 1:**
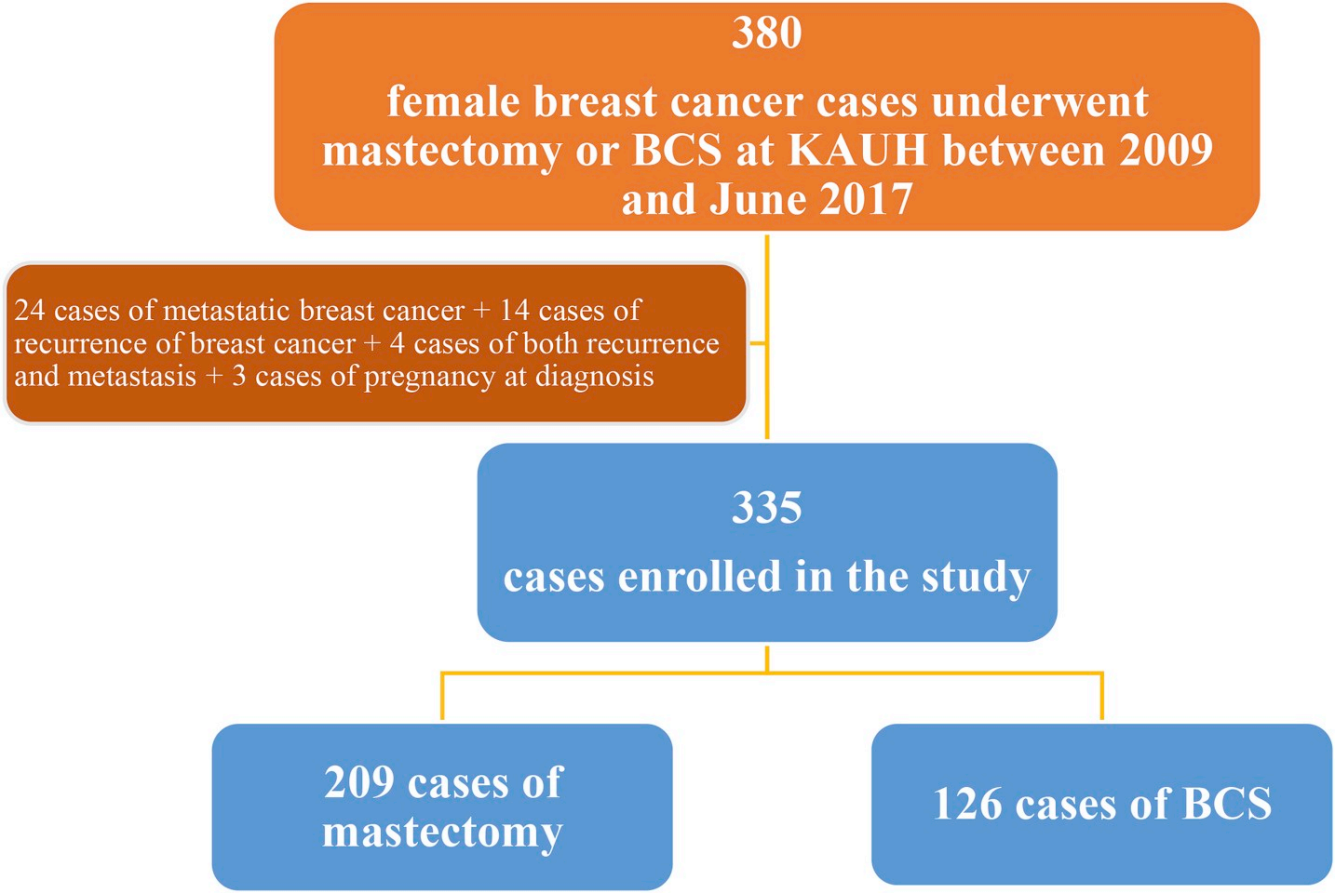
Flow diagram for the process of patients' enrollment in the study.

The data of 380 female patients who were diagnosed with breast cancer and underwent either mastectomy or BCS during the period between 2009 and June 2017 were collected. In total, 45 patients were excluded from the study; 24 of them had metastasis, 14 had recurrences, 4 had recurrence and metastasis, and 3 patients were pregnant at the time of diagnosis (Fig. 1).

**Fig. 2:**
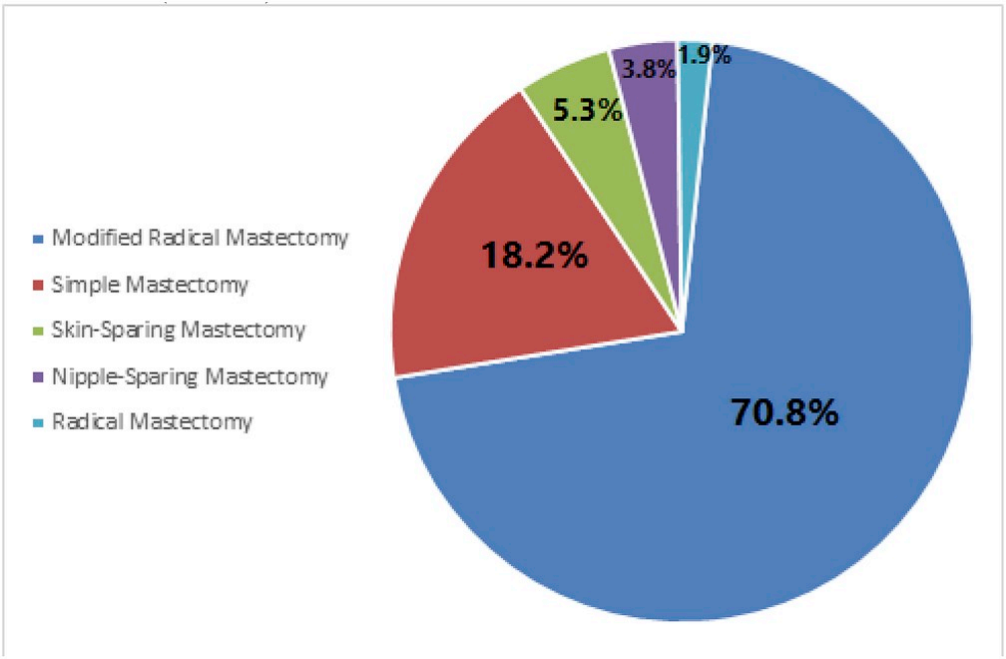
The percentages of each type of mastectomy out of all mastectomies (n = 209).

A total of 335 patients were enrolled in the study, and their ages ranged from 28 to 85 years old (mean ± SD: 52.75 ± 12.2 years). Of the total 335 patients, 209 (62.4%) underwent mastectomy, while 126 (37.6%) patients underwent BCS. The distribution of the patients' age groups and nationalities is shown in Table (1). In both groups, most of the patients were in the age group from 40 to 60 years old and were non-Saudi. Modified radical mastectomy was the dominant type of mastectomy (148 cases, 70.8%) followed by simple mastectomy (38 cases, 18.2%), skin-sparing mastectomy (11 cases, 5.3%), nipple-sparing mastectomy (8 cases, 3.8%), and radical mastectomy (4 cases, 1.9%) (Fig. 2).

**Table 1:** The correlation between demographic characteristics and the type of the procedure using Chi-square and independent T-tests (n = 335).

Characteristics	Mastectomy (N = 209, 62.4%)	Lumpectomy (N = 126, 37.6%)	*P* value
**Age** (years)	53.22 ± 12.74	51.97 ± 11.14	0.346
**Age groups**			0.107
<40 years	24 (11.5%)	12 (9.5%)	
40–60 years	126 (60.3%)	90 (71.4%)	
>60 years	59 (28.2%)	24 (19%)	
**Nationality**			**0.002**
Saudi	61 (29.2%)	58 (46%)	
Non- Saudi	148 (70.8%)	68 (54%)

The aim of the current study was to determine trends of mastectomy and breast-conserving surgery and to study the possible clinicopathological and radiological factors that may contribute to the decision making of the type of surgery to be performed.

Mastectomy was significantly more prevalent when the tumor appeared in more than one focus (multifocal) or extended to more than one quadrant (multicentric) (P = 0.0001). Mastectomy was also correlated with large-sized tumors (3.8 cm ± 2.75 cm), whereas BCS was correlated with the smaller ones (2.7 cm ± 1.75 cm) (P = 0.0001).

Furthermore, BCS was more prevalent in the patients who underwent preoperative breast MRI (39.3%) compared with those who did not (32.1%); this difference was significant (P = 0.001). Patients who underwent NACT (121 patients) were more likely to undergo a mastectomy (84 patients, 40.2%) than BCS (37 patients, 29.4%), P = 0.0001. Regarding the type of lymph node intervention, among the cases who had an axillary dissection, 153 cases (73.2%) had mastectomy, while 59 cases (46.8%) had BCS. On the other hand, the 83 cases that had sentinel lymph node biopsy (SLNB) or the 40 cases without lymph node intervention were more likely to have BCS (46 cases with SLNB [36.5%] and 21 cases without lymph node intervention [16.7%]) (P = 0.0001). The most common presenting stage was stage III (130 cases, 38.8%), followed by stage II with a slightly lower percentage (124 cases, 37%). Advanced stage, especially IIIA or more (94 cases, 47.4% of mastectomies) (P = 0.0001), and the presence of lymphovascular invasion or perineural invasion (P = 0.02) was more strongly linked to mastectomy than BCS. Tumor pathological type, histological (Nottingham) grading, and estrogen or progesterone receptor status separately did not have an effect on the decision of which procedure was chosen. However, HER2 receptor-positive status was correlated with mastectomy (P = 0.031), and triple-negative cases were correlated with BCS (P = 0.012).

By using multivariate analysis, the factors that were predictors for mastectomy over BCS were as follows: non-Saudi nationality (OR, 2.069; 95%CI, 1.306 to 3.278; P = 0.002); receiving neo-adjuvant chemotherapy (OR, 1.616; 95%CI, 1.008 to 2.593; P = 0.046); tumor with multifocality and multicentricity (OR, 2.557; 95%CI, 1.522 to 4.295; P = 0.0001 and OR, 4.155; 95%CI, 2.142 to 8.061; P = 0.0001); tumor size (OR, 1.247; 95%CI, 1.115 to 1.395; P = 0.0001) especially if the tumor size was above 5 cm (OR, 2.124; 95%CI, 1.166 to 3.868; P = 0.014); tumor stage IIIA and IIIB (OR, 2.753; 95%CI, 1.360 to 5.575; P = 0.005 and OR, 3.897; 95%CI, 1.317 to 11.528; P = 0.014); triple negative receptor status (OR, 2.109; 95%CI, 1.195 to 3.723; P = 0.010); and HER2 positive receptor status (OR, 1.963; 95%CI, 1.181 to 3.264; P = 0.009) (Table 2).

**Table 2:** Results of multivariable logistic regression models for the log odds of undergoing mastectomy as opposed to lumpectomy.

Variables	Odds Ratio	95%CI	P
**Nationality**			
Saudi	0.483	0.305–0.766	**0.002**
Non- Saudi	2.069	1.306–3.278	**0.002**
**Age Groups**			
<40 years	1.232	0.593–2.560	0.575
40–60 years	0.607	0.377–0.977	**0.040**
>60 years	1.672	0.977–2.860	0.061
**NACT**			
**Yes**	1.616	1.008–2.593	**0.046**
**No**	0.619	0.386–0.993	**0.046**
**Laterality**			
Right	1.042	0.669–1.622	0.856
Left	0.960	0.616–1.495	0.856
**Tumor Focality**			
Unifocal	0.391	0.233–0.657	**0.0001**
Multifocal	2.557	1.522–4.295	**0.0001**
**Tumor Centricity**			
Unicentric	0.241	0.124–0.467	**0.0001**
Multicentric	4.155	2.142–8.061	**0.0001**
**Tumor Size** (cm)	1.247	1.115–1.395	**0.0001**
**Tumor Size Groups**			
<2 cm	0.623	0.384–1.012	0.056
2–5 cm	0.936	0.601–1.456	0.768
>5 cm	2.124	1.166–3.868	**0.014**
**Tumor Stage**			
0	0.691	0.327–1.459	0.333
IA	0.259	0.126–0.529	**0.0001**
IIA	0.945	0.555–1.608	0.833
IIB	0.553	0.305–1.003	0.051
IIIA	2.753	1.360–5.575	**0.005**
IIIB	3.897	1.317–11.528	**0.014**
IIIC	2.060	0.900–4.714	0.087
**Tumor Grade**			
I	1.017	0.569–1.818	0.955
II	0.980	0.620–1.549	0.933
III	0.994	0.632–1.565	0.981
Unknown	1.059	0.431–2.600	0.900
**Positive Estrogen Receptor Status**	1.422	0.878–2.302	0.163
**Positive Progesterone Receptor Status**	1.145	0.723–1.813	0.656
**Positive HER2 Receptor Status**	1.963	1.181–3.264	**0.009**
**Triple Negative Tumors**	2.109	1.195–3.723	**0.010**
**Doing MRI**^a^	0.729	0.426–1.247	0.240
**MRI**^a^**Finding**			
Unilateral Malignant Findings	0.855	0.475–1.539	0.602
Bilateral Malignant Findings	1.283	0.607–2.709	0.514
Benign Findings	1.001	0.448–2.235	0.999

a MRI = magnetic resonance imaging.

## 4 Discussion

Our study revealed that the prevalence of mastectomy is higher than BCS in the surgical treatment of breast cancer, which is reversed from the pattern in the US and Europe over the years, with 13.1% up to 40.1% BCS vs 62.4% mastectomies [13,14,[28], [29], [30]]. This can be attributed to the relatively advanced stage at which most of the patients presented in this study.

The mean age in our study was 52.75 ± 12.16 years, which is slightly older than what Saggu et al. found (49.8 years) using data from the Saudi Cancer Registry between 1990 and 2010 [31] and other studies in Saudi Arabia [5,6], although most patients remained in the middle-age category. This average age is still approximately 10 years below the averages in the US and Europe [13,30]. The correlation with increased age and mastectomy is well documented in the US [13,28], but the extremes of age (<40 and ≥ 70) are both shown to be related to mastectomy by Garcia-Etienne et al. [30]. In our study, the only significant correlation was found between the age group 40–60 and BCS.

It is worth mentioning that in our study, the mastectomy rate, ages, and stages at presentation are comparable with the numbers in Arab countries found in 2007 by El Saghir et al. [32]. Noticeably higher rates of mastectomy are observed in other countries (up to 88% in Syria).

Nationality was found to be significantly related to the choice of surgery in our study. Non-Saudis had a significantly higher risk of going through a mastectomy. This can be attributed their limited health care access compared to Saudis at governmental hospitals as well as lower education in some cases, which both lead to late presentation and diagnosis.

Regarding factors affecting the choice of either type of surgery (mastectomy or BCS), most of our findings were consistent with the existing literature. Multifocality, multicentricity [30], larger tumor size [17,28], and more advanced lymph node status [30] and stage [17,29] are all linked to higher rates of mastectomy. However, the tumor's pathological type and grade were not significantly correlated with either surgery in our study, which contradicts the previously proposed correlation [28,30]. This finding might be due to the different distribution of pathological types in different samples and our patients predominantly having invasive ductal carcinomas. In this study, perineural and lymphovascular invasion and HER2 receptors were significantly correlated with mastectomy, while triple-negative breast cancer (TNBC) status was significantly correlated with BCS. In this respect, Garcia-Etienne et al. [30] reported a correlation between negative hormone receptor status and mastectomy. Preoperative MRI was shown to affect the choice of surgery although not always consistent towards either type of surgery [19,20]. In our study, it was significantly correlated with doing BCS rather than a mastectomy, a pattern that is found but not significantly correlated by Killelea et al. [20]. However, on multivariate analysis, the MRI findings were not significantly correlated with mastectomy, although it might be contributed as protective against mastectomy. Further investigation is required to determine whether there is a trend towards statistical significance or if there is a strong relationship between the result of the preoperative MRI and the choice of surgery.

NACT is a known factor that decreases the rate of mastectomy and increases the rate of BCS by downstaging the tumor [17]. However, we found in our study that it was a predictor of mastectomy and can be explained by different causes, such as lack of excellent response, lack of patient compliance with the chemotherapy, multicentricity of a tumor, or it may be due to other factors not studied, such as patient or surgeon preference. All of these factors could not be assessed in this retrospective study. This also may be explained by patients being in advanced stages and with large-sized tumors at the time of diagnosis, which could be related to mastectomy. This could also open the question of whether the expected relieving effect of NAT is reached, well-measured, or is taken into account when planning for surgery.

Most of the patients who underwent a mastectomy in our study had modified radical mastectomy, and only a small percentage underwent conservative mastectomy. Planning breast reconstruction after mastectomy is a known factor in preferring one type of surgery to the other, although it is not adequately discussed with patients [18]. However, only a few patients in our study had breast reconstruction (32 patients, 9.55%), which reduced the ability to reliably examine a relationship.

Breast size is another important factor in preferring BCS or mastectomy, but it was not examined in this study since it was not documented in the medical record. Nonetheless, breast size is implicated in the choice of surgery type and should be taken into account in studying the factors affecting such a decision, as larger breasts may be able to reserve some volume despite complete excision of a tumor.

In our current study, some factors could not be assessed, including patients' choice, family history, and surgeon's experience and level of training. These factors were poorly documented in addition to incomplete clinical, pathological and radiological documentations, resulting in the inability to study some important factors used to choose between mastectomy and BCS, especially in older cases due to the relatively late introduction of the electronic filing system to the hospital. This has been observed also by Yousef et al. in 2004 at King Faisal Specialist Hospital in Riyadh [33].

## 5 Conclusion

In our study, mastectomy procedures have a much higher prevalence than in the developed world. Some are due to relative indications, but most of the cases in the gray area where the doctor's and the patient's opinions play an important role in the decision-making process. These correlations between the factors that have been supported by existing literature emphasize the importance of early detection of breast cancer to move further in the direct of breast-conserving therapy, especially in our area, for improved and better-tolerated outcomes. The significant correlation of mastectomy with non-Saudis sounds an alarm with respect to further efforts to ensure wider healthcare access, education and advocacy for early detection of breast cancer. We recommend further studies be carried out to examine other factors that are important for decision making, such as breast size, planning of breast reconstruction after mastectomy, and patient and surgeon preferences.

## Conflicts of interest

The authors declare that they have no conflicts of interest. Regarding financial details, we have received no support or commercial funding for this study.

## Ethical approval

Ethical approval was obtained from the Research Ethics Committee of the Faculty of Medicine, King Abdulaziz University (Reference No. 357–17).

## Author contribution

Zuhoor K. Al-Gaithy: Conceptualization, Methodology, Validation, Formal Analysis, Writing – Review & Editing, Supervision, Project Administration.

Bassam E. Yaghmoor: Investigation, Data Curation, Writing – Original Draft, Project Administration.

Mohammed I. Koumu: Investigation, Writing – Original Draft.

Khalid A. Alshehri: Investigation, Writing – Original Draft.

Abed A. Saqah: Investigation, Writing – Original Draft.

Hisham Z. Alshehri: Investigation, Writing – Original Draft.

## Conflicts of interest

There are no conflicts of interest.

## Trial registry number

ClinicalTrials.gov.

UIN: 357-71.

ClinicalTrials.gov Identifier: NCT03762642.

https://clinicaltrials.gov/ct2/show/NCT03762642.

## Guarantor

Zuhoor K. Al-Gaithy, Bassam E. Yaghmoor.

## Peer review and provenance

Not commissioned, externally peer reviewed.

## Sources of funding

Our research received no funding.
